# High Antibody-Dependent Cellular Cytotoxicity Responses Are Correlated with Strong CD8 T Cell Viral Suppressive Activity but Not with B57 Status in HIV-1 Elite Controllers

**DOI:** 10.1371/journal.pone.0074855

**Published:** 2013-09-23

**Authors:** Olivier Lambotte, Justin Pollara, Faroudy Boufassa, Christiane Moog, Alain Venet, Barton F. Haynes, Jean-François Delfraissy, Asier Saez-Cirion, Guido Ferrari

**Affiliations:** 1 INSERM, U1012, Bicêtre, France; 2 AP-HP, Department of Internal Medicine and Infectious Diseases, Bicêtre Hospital, Bicêtre, France; 3 University Paris-Sud, Bicêtre, France; 4 Duke Human Vaccine Institute, Departments of Medicine and Surgery, Duke School of Medicine, Durham, North Carolina, United States of America; 5 INSERM CESP U1018, Bicêtre, France; 6 INSERM U778, Strasbourg, France; 7 Regulation of retroviral infections Unit, Institut Pasteur, Paris, France; University of Cape Town, South Africa

## Abstract

The role of Antibody-dependent cellular cytotoxicity (ADCC) responses in HIV-1 controllers is still unclear due to the heterogeneity of these patients. We analyzed 67 HIV-1 controllers and found significantly higher levels of ADCC antibodies in controllers versus viremic subjects (p = 0.017). Moreover, multivariate analysis revealed significantly higher ADCC titers in HLA B57- controllers compared to HLA-B57+ ones (p = 0.0086). These data suggest a role for ADCC in immune control of HIV, especially in HLA B57 negative controllers.

## Introduction

The immune response required for protection from HIV infection in humans has not been fully defined but will likely involve both strong cellular and humoral immunity. Antibody-dependent cellular cytotoxicity (ADCC) is of special interest since this mechanism has been suggested to play a role in the RV144 vaccine trial [1], and because several studies have associated the ADCC activity of sera with slow clinical progression and protection from mother-to-infant transmission [2], [3]. Moreover, a recent rhesus passive protection study has shown the importance of Fc Receptor (FcR)-dependent antibody (Ab) functions in mediating protective anti-SHIV activities [4].

Rare HIV-1-infected patients, termed HIV controllers (HIC), maintain plasma HIV RNA levels below the limit of detection for a prolonged period of time without therapy [5], [6]. Solid data support the role of cellular immunity for controlling HIV replication in a large fraction of HIC including the overrepresentation of the HLA allele B*5701 [5], [6], a strong HIV-specific CD8 T cell response with HIV-suppressive activity [5], [7], and preservation of central memory CD4 T cells [8].

The involvement of humoral immunity in the control of HIV replication in HIC is still unclear, but non-neutralizing Abs are candidates to play a role. In fact, studies conducted by our group and others indicated the presence of higher ADCC titers in HIC compared to viremic subjects [9], [10]. Antibody-dependent cellular viral inhibition was also found to be higher in HIC than in viremic patients [11]. However, ADCC results were collected from a small number of patients with limited representation of the variety of controllers with particular regards to expression of HLA-B57 alleles.

In this study, we analyzed ADCC responses in the first 67 HIC enrolled in the French ANRS HIV Controller cohort and compared to those detectable in 40 patients who could not control virus replication. We found significantly higher levels of ADCC antibodies in controllers versus viremic subjects. In addition, the presence of HLA-B57+ (49%) and HLA-B57- (51%) among the HIC enabled us to perform multivariate analysis to identify immune activities associated with high ADCC titers. We found that ADCC titers were significantly higher in HLA B57- controllers compared to HLA-B57+ controllers (p = 0.0086).

## Patients and Methods

### Ethics statement

All the subjects gave written informed consent to the study and the ethical committee of Bicêtre Hospital (Comité de Protection des Personnes Ile de France VII, n°05–22) and the Institutional Review Board of Duke University approved the studies performed.

### Patients

HIV controllers consecutively enrolled in the ANRS CO18 HIV Controller cohort were selected on the basis of the following characteristics: HIV-1-infected subject with a follow-up longer than 5 years, without any antiretroviral treatment, and with the five last plasma HIV RNA measurements lower than 400 copies/mL (Table 1). Controllers were classified either on the HLA B57 status, or on the ability of their CD8^+^ T cells to suppress viral replication in CD4:CD8 cocultures as previously published [5]. The suppression of viral replication was calculated as the logarithm of the decrease of p24 production in the coculture (log10 p24 decrease). This assay allowed us to discriminate Strong CD8 Responders (SR), with strong CD8 T cell ability to block viral replication (log10p24 decrease≥2) and Weak CD8 Responders (WR), with a lower ability to block viral replication (log10p24 decrease<2) [5]. Fifty two percent of HIC were SR and 48% WR. Among B57+ controllers, 48% were SR whereas among B57- HIC, 54% were SR. IFN-γ-producing HIV-specific CD8 T cells were quantified by ELISPOT assay (median 1960 SFCs (IQR 665-4200) using a set of peptides corresponding to known optimal HIV-CTL epitopes (NIH HIV Molecular Immunology Database: http://www.hiv.lanl.gov/content/immunology/tables/optimal_ctl_ summary.html) according to the subjects' HLA type, as previously described [5]. Ultrasensitive plasma viral RNA levels (threshold 4 RNA copies/mL) were not significantly different between B57+ and B57- HIC, or between SR and WR.

**Table 1:** Characteristics of HIV controllers and viremic untreated patients.

	HIV controller (n = 67)	Viremic patients (n = 40)
Median age (years)/male	44 [IQR 40–50]/50%	40 [IQR 34–50]/62%
Median of CD4+ T cell count (/mm3)	755 [IQR 554–951]	466 [IQR 325–561]
Median of viral DNA (log_10_ copies/million PBMCs)	1.48 log10 [IQR 1.34–1.91]	Not available
Median of plasma HIV RNA (log_10_ copies/mL)*	1.42 log10 [IQR 0.6–1.9]	4.5 log10 [IQR 4.2–4.9]

* quantified with ultrasensitive test.

As a control group, 40 viremic chronically-infected untreated patients were randomly selected at the Infectious Diseases Department of University Bicetre Hospital and plasmas collected. All the viremic patients tested were B57 negative except one.

### Virus, Infectious molecular clones (IMC) for ADCC GTL assay

HIV-1 reporter virus used was replication-competent IMC designed to encode the BaL (subtype B) env gene in cis within an isogenic backbone that also expresses the Renilla luciferase reporter gene and preserves all viral open reading frames, NL-LucR. T2A-BaL.ecto (IMC_BaL_) [12]. Reporter virus stocks were generated by transfection of 293T cells with proviral IMC plasmid DNA and titered on TZM-bl cells for quality control.

### ADCC-GTL assay

Antibody Dependent Cellular Cytotoxic (ADCC) activity was detected according to our previously described ADCC-GranToxiLux (GTL) procedure using HIV-1 IMC_BaL_-infected CEM. NK_RCCR5_ (ADCC-BaL) as target cells and cryopreserved PBMC from a HIV-seronegative donor as effector cells [13]. The results of the GTL assay were considered positive if % Granzyme B activity after background subtraction was ≥8% for the infected target cells as determined during the standardization of our assay [12]. The log_10_ titer of the ADCC antibodies present in the plasma was calculated by interpolating the log_10_ reciprocal of the last plasma dilution that yielded positive % Granzyme B activity (≥8%).

### Statistical analysis

ADCC, HIV-1 RNA and HIV-1 DNA were log_10_-transformed instead of using the raw values. All comparisons were made using non-parametric tests Since data did not fulfill classic linear model assumptions, non-parametric bootstrap linear regression models were used in univariable and multivariable models. Only variables with a p<0.20 in univariate regression analysis were included in the final multivariate model. STATA programs (Version 12.1, 2011; Stata Corp., College Station, Texas) were used for statistical analysis.

## Results

**Figure 1:**
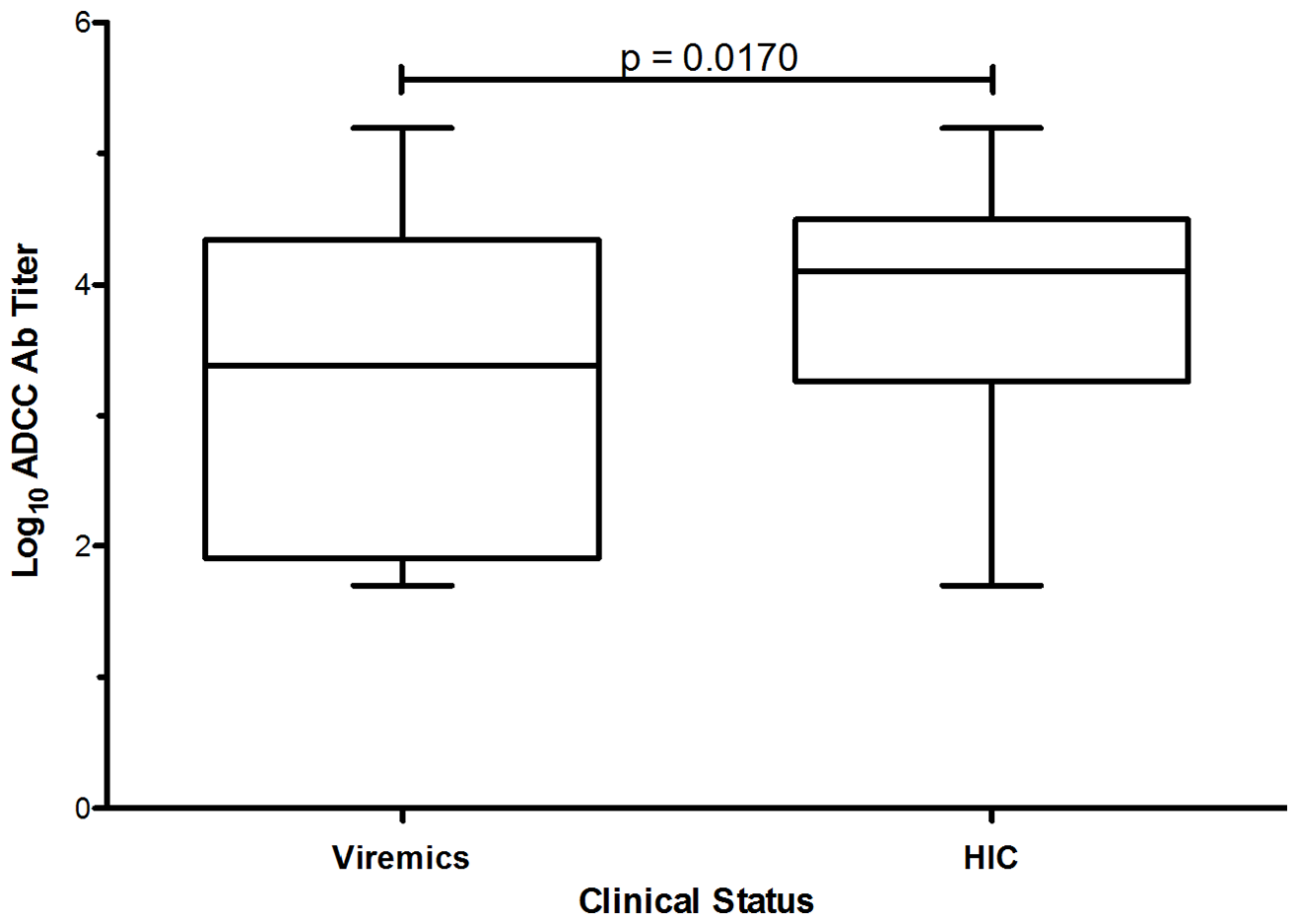
ADCC responses in HIV-1 Viremic and Controller (HIC) patients. The whisker plots represent the distribution of Log_10_ ADCC Ab titers among the 40 viremic and the 67 HIC patients. ADCC activity was detected according to our previously described ADCC-GranToxiLux (GTL) procedure [13].

ADCC responses were detected in the plasma of 61 HIC (90% detectable ADCC), but only in 32 of the viremic patients (82%). We observed a statistically significant higher median titer of ADCC in the Controllers compared to the Viremics (p = 0.017, Figure 1). Among the six HIC with undetectable ADCC responses, five were “elite” controllers with undetectable RNA during all their follow up (more than 10 years), contrasting with 18 “elite” among the 61 HIC with detectable ADCC (p = 0.02). Thus, HIC had higher log_10_ ADCC titers than viremic patients. This led us to investigate whether ADCC correlates with other parameters defining HIV-1 infection using multivariate analysis.

Since HIC is a heterogeneous group, we looked for relationships between log_10_ ADCC titers and other variables: sex, ethnicity, HLA B27 and B57 status, the time from HIV diagnosis, CD4 T cell count, plasma viral RNA, cellular viral DNA, the number of HIV-specific CD8 T cells (total SFC), the CD8 T cell ability to suppress viral replication (log10 p24 decrease), and the Strong Responder/Weak Responder status. In the univariate analysis, plasma viral RNA, virus suppression activity (log_10_ p24 decrease), and time from HIV diagnosis were significantly positively correlated with log_10_ ADCC titers (respectively, r = 0.32 and p<0.001, r = 0.44 and p<0.001, and r = 0.35 and p = 0.003 (spearman rank correlation)). In contrast, log_10_ ADCC titers were lower in females versus males (p = 0.04), in WR versus SR (p = 0.02) and in B57+ patients versus B57- (p = 0.02). Other variables were not related with log_10_ ADCC (Table 2).

**Table 2:** Univariate and multivariate bootstrap linear regression analysis of the role of age, sex, HLA B57, Delay since diagnosis, and the CD4 T cell counts or HIV-1 RNA levels in log_10_ ADCC in the 67 HIV-infected patients enrolled in the ANRS CO18 cohort.

	Univariate		Multivariate	
Characteristics	β coefficient	p value	β coefficient	p value
Weak responders	−0.57	0.02	−0.53	0.005
Female sex	−0.44	0.04	−0.06	0.78
HLA B57 positive	−0.53	0.02	−0.47	0.0086
Delay since diagnosis*	+0.05	0.003	+0.06	0.001
HIV-RNA (log_10_ copies/mL)**	+0.58	<0.001	+0.57	<0.001
CD4 cell count (/mm^3^)***	−0.05	0.17	−0.003	0.75
Ehnicity	−0.003	0.99	-	-
HLA B27 positive	+0.09	0.82	-	-
HIV-DNA (log_10_ million PBMCs)	+0.29	0.36	-	-
Number of HIV-specific CD8 T cells (SFC) ****	+0.72	0.007	-	-

The multivariate analysis included all variables.

* per one year increase; ** per a 1 log copies/mL increase, ***per a 100-CD4 increase;. Weak responders were compared to Strong responders; females were compared to males, HLA B57 positive patients to HLA B57 negative patients, White patients were compared with others, HLA B27 positive patients to HLA B27 negative patients. **** The number of HIV-specific CD8 T cells was not included in the multivariate analysis because there was a strong link with the Weak/Strong Responders status: all the Weak Responders had SFC below the median (less than 1960 SFC).

**Figure 2:**
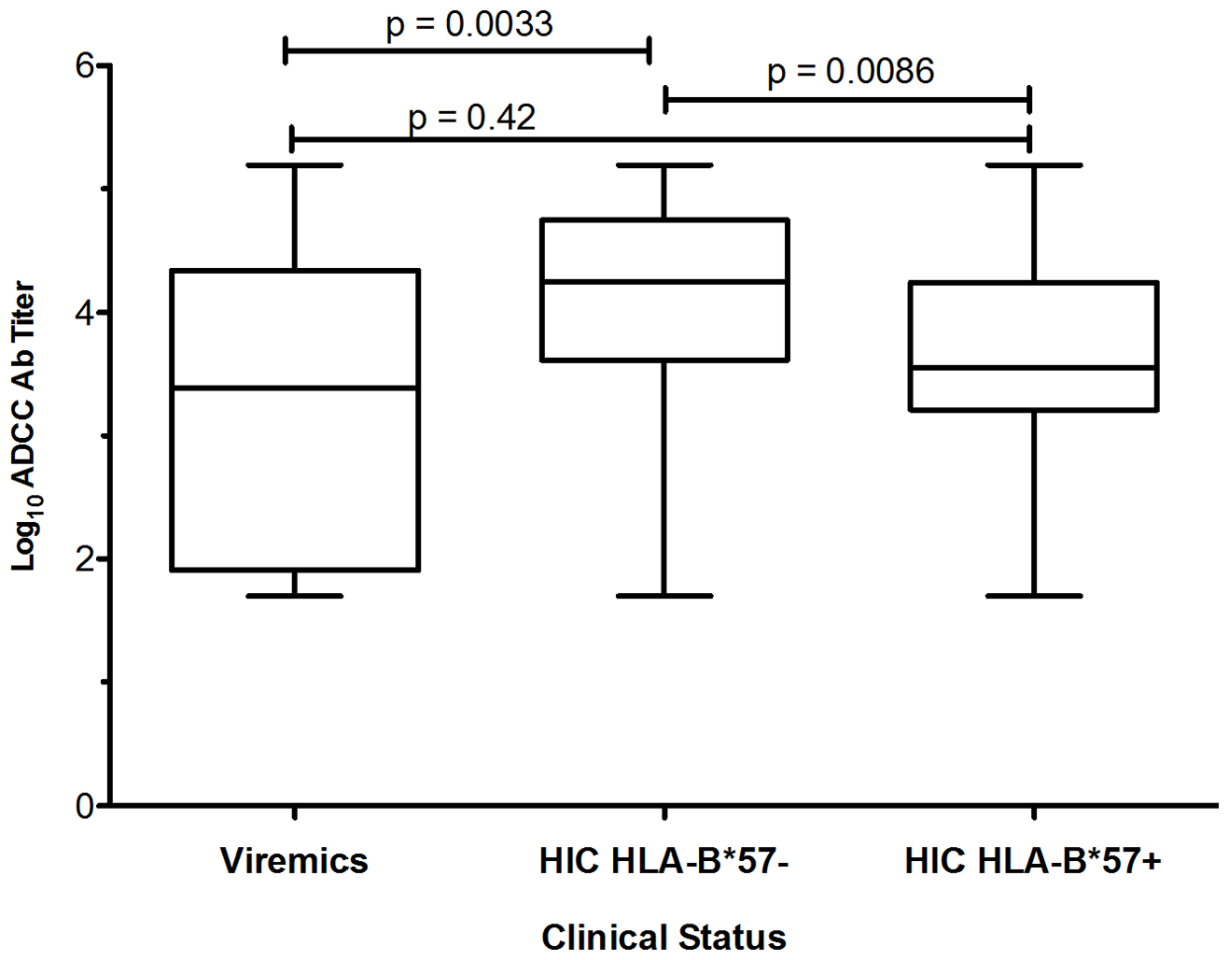
The whisker plots represent the distribution of Log_10_ ADCC Ab titers among the viremic and the HIC patients, separated according to B57 status.

“Multivariate statistical analysis was therefore performed (Table 2). Log_10_ ADCC titers remained positively correlated with time from HIV diagnosis (p = 0.001), plasma HIV RNA (p<0.001), and with the Strong Responder status (p = 0.005) in HIC. Log_10_ ADCC titers were significantly higher (p = 0.0086) in B57- patients compared to B57+ ones (Table 2 and Figure 2).

B57- HIC patients had also significantly higher log_10_ ADCC titers than viremic patients (p = 0.0033), contrasting with B57+ HIC in whom ADCC titers were not different compared with viremic patients.”

**Figure 3:**
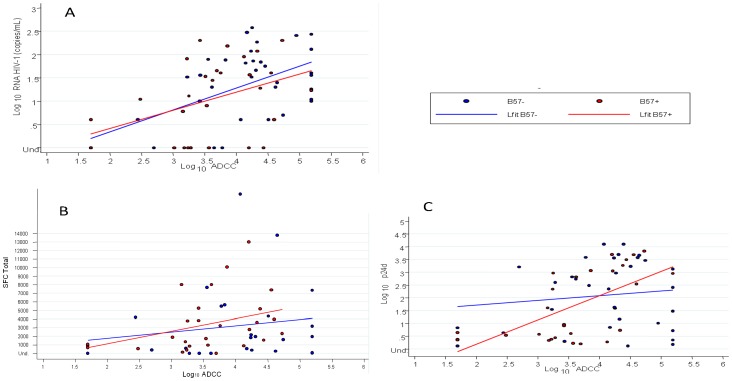
Scatterplot with overlaid linear prediction plot between the log_10_ ADCC titers and (A) the log_10_ RNA HIV-1 viral load (r = 0.37 and p = 0.03 in B57-, r = 0.43 and p = 0.02 in B57+), (B) the number of HIV-specific CD8 T cells (r = 0.21 and p = 0.31 in B57-, r = 0.44 and p = 0.02 in B57+), or (C) the ability of CD8 T cell to control viral replication quantified by the log_10_ p24 decrease (r = 0.02 and p = 0.93 in B57-, r = 0.60 and p = 0.0008 in B57+), respectively in HLA B57+ (red) and HLA B57- controllers (blue).

Multivariate analysis was then performed on B57+ and B57- HIC subgroups. In B57+ HIC, there were positive correlations between log_10_ ADCC titers and plasma viral RNA, and CD8 viral suppressive capacity but also with the number of IFNγ-producing HIV-specific CD8 T cells (Figure 3). This last result was consistent with our previous data showing a correlation between the number of HIV-specific CD8 T cells and the ability of CD8 T cell to control viral replication [5]. In contrast, log_10_ ADCC titers did not correlate with any parameter in B57- HIC, except with the plasma viral load (p = 0.03) (Figure 3).

## Discussion

We show here that HIC had higher log_10_ ADCC titers than viremic patients. This result is in accordance with Johansson's study [10] and also expands our previous work [9] with the highest number of HIC studied to date. It also fully supports recent data showing strong ADCVI in controllers [11]. Interestingly, in a recent work focusing on long-term slow progressors including 25% of HIV controllers, significantly broader ADCC responses were found in these patients compared with progressors, with antibodies targeted specifically regulatory/accessory HIV-1 proteins [14]. The number of HIC studied here allowed for better representation of the heterogeneity in HIC according to their ADCC titers. This led us to look for correlates of ADCC using multivariate analysis.

ADCC was higher in patients with strong CD8 suppressive activity (Strong Responders) compared to WR. This result may represent indirect evidence of efficacious CD4 T cell help among these patients as T cell help drives the development of both humoral and cell-mediated immunity. Another possible explanation is that cellular mediated suppressive activity reduces the level of immune activation and thereby influences the profiles of Ab glycosylation that influence FcR-binding Ab functions such as ADCC [11], [15], [16]. These findings are in agreement with the most recent observations by Ackerman and collaborators [11].

In HIC, ADCC was also positively correlated with plasma HIV RNA. This suggests that ADCC may be subjected to stimulation by viral replication. However, we found that ADCC titers were significantly lower in viremic patients despite higher viral loads than HIC. This dichotomy suggests that the development of sufficient amounts of ADCC Abs requires adequate levels of CD4 help in presence of antigen stimulation to maintain efficient HIV-specific Ab producing B cells. Functional HIV-specific CD4 T cells are present in HIC [8] and the B cell compartment seems to be preserved [17], whereas these two requirements are lacking in viremic patients [18]. We found that HIC who had undetectable ADCC levels were mainly “elite” controllers who did not have any viral RNA in plasma above the detection thresholds of the commercially available assays. In these patients, the limited amounts of viral antigens could be insufficient for maintaining detectable ADCC titers. The very low viral replication seen in HIC and in patients on antiretroviral treatment (ART) could be one of the stimuli necessary for plasma cells to produce high titers of protective agalactosylated glycoforms which have been involved in strong ADCVI [11].

The necessity to have a very low viral replication to maintain high ADCC Ab titers is further supported by the positive correlation between ADCC and the time from HIV diagnosis. A recent rhesus monkey study also supports this result: in an attenuated SIV model with low grade replication, ADCC titers increased with time and broadening of antibodies specificities [19].

Lastly, we investigated the role of HLA-B*57 expression. Multivariate analysis was performed on B57+ and B57- HIC subgroups. In B57+ HIC, there were positive correlations between log_10_ ADCC titers and, respectively, plasma viral RNA, and CD8 viral suppressive capacity but also with the number of IFNγ-producing HIV-specific CD8 T cells (Figure 3). This last result was consistent with our previous data showing a correlation between the number of HIV-specific CD8 T cells and the ability of CD8 T cell to control viral replication [5]. This suggests that both cellular and humoral immunity share an important role for controlling HIV within the B57+ HIC. The lower ADCC titers we observed in HLA B57+ versus B57 negative controllers are in agreement with results previously published in a study in which 6/9 HIC were B57+ [20]. Our work therefore reconciles previous published data on ADCC in HIC, but underscores the heterogeneity of these patients. Indeed, log_10_ ADCC titers did not correlate with any parameter in B57- HIC, except with the plasma viral load (p = 0.03) (Figure 3). This suggests that in these patients, ADCC could be one of the major effective immune mechanisms that contribute to the control of virus replication during the course of natural infection. These patients are probably those who are the most interesting to study further for insight into the protective features of ADCC. Interestingly, a recent study has also pointed out stronger protective Ab titers in HIC without protective HLA alleles compared with B57+ ones [21]. At this time, it is unclear why high ADCC titers or higher HIV-specific IgG2 [21] would be differentially induced only in controllers lacking B57 alleles. There was no difference in the ADCC titers in B27+ and B27- HIV controllers but the low number of HLA B27+ controllers was a limit of the analysis.

In conclusion, ADCC was significantly higher in HIC than in viremic non-controllers. In addition, this study is the first to identify differences in ADCC activity among HIC according to HLA-B57 status. The correlations found indicate that ADCC could play a role in B57- HIC. Thus, this finding suggests that eliciting ADCC responses could be an important objective for a vaccine as most of the general population is B57-. However, the specific characteristics of the ADCC Abs in B57- HIC including the epitopes targeted, Fc glycosylation, and interactions with FcγRs have yet to be described. Understanding these characteristics will be essential to inform new strategies and targets for the induction of such Abs by vaccination. In addition, to further investigate the role of ADCC in HIC it will be key to test ADCC-mediating, non-neutralizing antibodies in non-human primate passive protection trials to directly determine their protective capacity.
